# Exploring Horizons in the Treatment of Vasoplegia in Shock Syndromes

**DOI:** 10.5152/eurasianjmed.2022.22094

**Published:** 2022-12-01

**Authors:** Ricardo Oliveira dos Santos Soares, Paulo Roberto Barbosa Evora

**Affiliations:** 1 Marília Medical School, Av. Monte Carmelo, Marília, Brazil; 2 Department of Surgery & Anatomy, Ribeirão Preto Medical School, University of São Paulo, Ribeirão Preto, Brazil

**Keywords:** Distributive shock, vasoplegia, vasopressors, microcirculation

## Abstract

Vasoplegic endothelial dysfunction stands out as one of the most prominent shock syndromes in the intensive care unit, and despite continual therapeutic advances, it is still associated with poor prognosis in critical cases. This scenario is compatible with a significant inflammatory disturbance, with a propensity for increased vascular permeability and deterioration of endothelial response to modulators: a microcirculation disaster. The hemodynamic support's backbone is based primarily on fluid replacement and the use of vasopressor and inotropic agents in nonresponsive patients, aiming to establish a mean arterial pressure of at least 65 mmHg and therefore promote adequate tissue reperfusion. The present study’s primary target is to discuss the combination of 3 concepts as a useful strategy for improving results against the high rates of mortality in critically ill patients. These 3 concepts are (1) the use of “broad-spectrum vasopressors,” (2) vasopressor-sparing strategy, and (3) microcirculation protection.

**Main Points**

- Acute myocardial ischemia, myocardial infarction, and sepsis are typical clinical settings of vasoplegia.
- This review discusses the interplay of 3 key concepts to keep in mind as a strategy to improve outcomes against the high mortality rates in critically ill patients: “broad-spectrum vasopressors,” vasopressor-sparing strategies, and microcirculation protection.
- “Sparing strategies” and “microcirculatory protection” have a pivotal role in avoiding the “broad spectrum vasopressor support.”

## Introduction

The most common etiologies of vasoplegic syndrome (VS) are sepsis and anaphylaxis and to a lesser extent, trauma, adrenal insufficiency, capillary leak syndrome, recreational drug overdose, and adverse effects of calcium channel blockers. The VS stands out as one of the most prominent shock syndromes in the intensive care unit, and despite continual therapeutic advances, it is still associated with poor prognosis in critical cases. The clinical scenario suggests a significant inflammatory disturbance, with a propensity for increased vascular permeability and deterioration of endothelial response to modulators.^2^ The hemodynamic support's backbone is based primarily on fluid replacement and the use of vasopressor and/or inotropic agents in nonresponsive patients, aiming to establish a mean arterial pressure (MAP) of at least 65 mmHg. However, in the underlying inflammatory state of shock, adrenoreceptor sensitivity decreases, and refractory patients may require high dosages of catecholamine to achieve hemodynamic stability. In this scenario, catecholamine toxicity arises, and the excess of adrenergic agents may cause direct organ damage, with detrimental outcomes to the immune system, metabolism, and coagulation pathways, contributing to a poorer prognosis.

It has been described that trauma patients who present with elevated epinephrine levels at admission are subjected to increased mortality. Johansson and colleagues (2012)^3^ identified through a prospective study that higher epinephrine levels in non-survivors were associated with biomarkers of tissue and endothelial damage, hypocoagulability, and hyperfibrinolysis. Therefore, it is imperative to revisit the established protocols of the high dosage of catecholamines with the risk of vasopressor-induced adverse events.

The study adopts the definition of distributive vasoplegic shock as a synonym of “vasoplegic endothelium dysfunction.”

The Jekyll-and-Hyde catecholamine conundrum analogy by Andreis and Singer (2016)^4^ brilliantly illustrates the harmonious synergy of these levels and also describes the iatrogenic over-activation in an attempt of shock stabilization, at the cost of microcirculation damage and a reasonable prognosis. It has been described that trauma patients with elevated epinephrine levels at admission are associated with biomarkers of tissue and endothelial damage, hypocoagulability, and hyperfibrinolysis.^3^ Therefore, it is imperative to revisit the established protocols for high-dosage catecholamines with the risk of vasopressor-induced adverse events by exploring complementary or alternative strategies. Fortunately, the medical literature has been increasingly flirting with non-catecholaminergic synergistic biochemical mechanisms that may allow for a more physiologically plausible dosage of catecholamines and have lesser adverse effects.^4,5^

**Figure 1.**
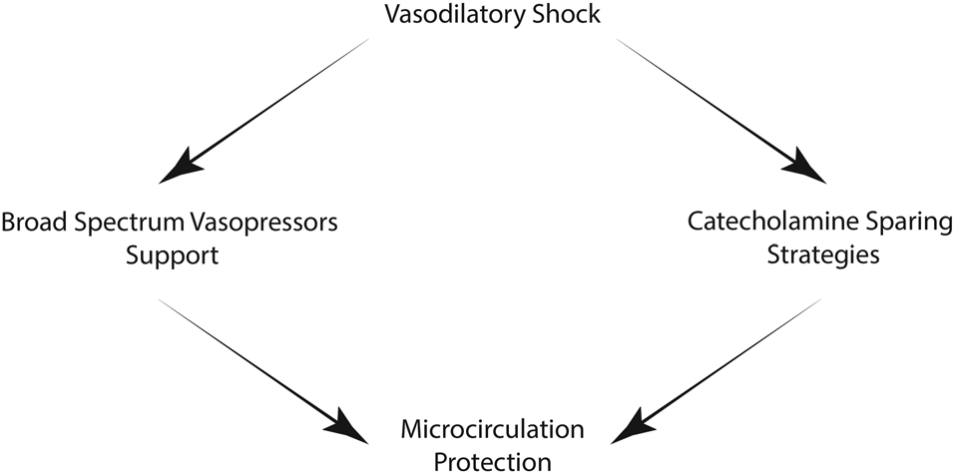
The overview of the desirable steps to better approach a critically-ill patient in the vasodilatory shock: (1) use of “broad-spectrum vasopressors,” (2) vasopressor-sparing strategy, and (3) microcirculation protection.

**Figure 2.**
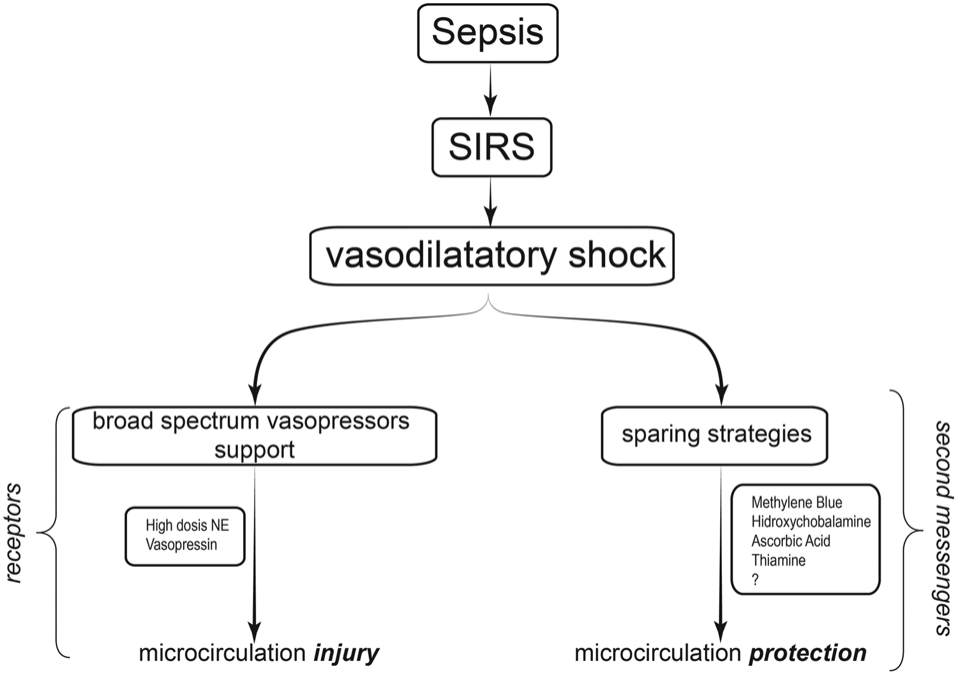
Schematic presentation showing that “sparing strategies” and “microcirculatory protection” have pivotal role to avoid the “broad spectrum vasopressors support.”

In this review, we discuss the interplay of 3 key concepts to keep in mind as a strategy to improve outcomes against the high mortality rates in critically ill patients: (1) “broad-spectrum vasopressors,” (2) vasopressor-sparing strategies, and (3) microcirculation protection (Figures 1 and 2).

## Typical Clinical Settings of Vasoplegia

Acute myocardial ischemia and infarction (MI) cause an overabundance of catecholamine release: epinephrine, by engaging the sympathoadrenal system and norepinephrine (NE), by direct myocardium secretion. When post-cardiac arrest syndrome occurs, the resulting ischemia-reperfusion injury leads to a pathological shedding of the endothelial glycocalyx, as identified by increased blood levels of syndecan-1, heparin sulfate, and hyaluronic acid: “a microcirculation disaster.”^6^

### Sepsis Vasoplegia

Sepsis drives profound microvascular alterations that may lead to grave consequences, such as generalized organ failure. Among several pathological conditions, the hyporesponsiveness of adrenergic stimuli by the smooth muscle lining of the arterioles deserves a unique glance regarding the resuscitation of shock patients. Apart from inadequate response from the sympathetic system’s endogenous catecholamines, the arterioles may only respond to exogenous vasopressors when in supraphysiological concentrations, if at all. This phenomenon is attributed to a multitude of complex mechanisms, which can be divided didactically into 2 categories: macro and microcirculation causes. Sepsis may induce macro dynamic changes in the balance of vasoactive agents in the blood such as vasopressin deficiency, characterized by diminished plasma levels; elevation of endothelin secretion by the heart, lungs, intestine, liver, and others; increase in vasodilator peptides (adrenomedullin and calcitonin gene-related peptide), and an increase in oxidative stress which intensifies catecholamines degradation and inactivation.^7,8^

The endothelium's hyporesponsiveness and the arterial smooth muscle lining of the microvasculature derive much from an impaired expression of vasoconstrictive receptors (angiotensin, adrenergic, and vasopressin receptors).^9^

### Pharmacologic Options

The 2018 edition of the Survival Sepsis Campaign (SSC) guidelines consider that a proper therapeutic approach to vascular hyporesponsiveness should count not only on catecholamine vasopressors regulation but also on non-catecholamine vasopressors, such as vasopressin.^1^ Notwithstanding, it is desirable to broaden the therapeutic options, to prevent excessive inflammatory response and also to make use of the synergistic access to vasopressor pathways that each compound may independently stimulate, as a broad-spectrum vasopressor concept.

### Catecholamine Vasopressors

Apart from the aforementioned complications when used in excessive concentrations, norepinephrine remains the drug of choice in the treatment of distributive shock and for several good reasons. It increases MAP without causing tachycardia, has vasopressor potency as high as epinephrine and phenylephrine (a sympathomimetic amine), and is higher than dopamine,^10,11^ does not promote the increase of lactate levels,^12^ and increases cardiac index both by selective α1/β1 stimulation in the myocytes and mobilization of splanchnic volume.^13^

Phenylephrine is a selective α1-adrenergic agonist that produces characteristic splanchnic vasoconstriction that could improve the cardiac index. However, its use as an exclusive vasopressor in septic shock has been found to increase in-hospital mortality compared to alternatives. Dopamine may lead to undesired hemodynamics and metabolic and inflammatory responses. Dopamine in septic shock could enhance the risk of the intrapulmonary shunt, leading to an increase in venous return. But it is a safer choice to be used in patients with previous myocardial disease. Because of its tachycardic and tachyarrhythmia adverse effects, Russel et al^14^ suggest that dopamine should be used when epinephrine or norepinephrine is not available. Considering all these parameters, Levy et al (2018)^15^ concluded that it is more logical and allows much better control of hemodynamics to use a separate titration of inotropic agent and vasopressor, such as dobutamine and NE, respectively, than using epinephrine alone.

### Non-catecholamine Vasopressors

#### Derivatives from the Renin-Angiotensin-Aldosterone System

Recent studies agree that the administration of ATII is a valid complementary step when the use of NE and AV1 produces unsatisfactory results toward the target MAP. The rationale here is based on reaching the hemodynamic goals while sparing deleterious effects of higher dosages of catecholamine vasopressors. Arginine vasopressin (AV1) is a hormone whose primary functions are vasoconstriction and hydric balance by modulating diuresis, exerting an antidiuretic effect. In VS, the AV1 can counteract the persistent opening of ATP-sensitive potassium channels (KATP) channels in smooth muscle cells, reverting the state of hyperpolarization and hyporesponsiveness of the membrane to catecholamines.^16^ Although AV1 presents all of its advantages and is also an endogenous hormone, experimental evidence suggests that synthetic selective AV1 agonists may offer superior outcomes. At a dosage of 2.5 ng/kg/minute, Russel et al (2017)^14^ showed that the infusion of selepressin (short-acting selective AV1 agonist) at early stages of VS is of rapid onset and is a lasting activity. It increased the proportion of patients who weaned off NE in the first 24 hours, therefore diminishing the mean cumulative dose of NE, while potentially lowering the time of intubation.^14^

Another synthetic vasopressin analog is terlipressin. Like selepressin, terlipressin has a selective affinity for AV1 receptors. However, contrary to the first, its effect is long-lasting. The literature on terlipressin is relatively new, and clinical data suggest that its use may be more beneficial in VS when compared to catecholamine monotherapy. One of the greatest gaps in terlipressin is that its effect on mortality in the treatment of VS is little known. Liu et al (2018)^17^ designed and carried out a multicenter, randomized, and double-blind trial and observed no statistical difference in mortality between the groups treated with an infusion of terlipressin or NE. The terlipressin group showed a higher number of serious adverse effects, with digital ischemia being the most common. More recently, Huang et al^18^ conducted a meta-analysis that concluded that the treatment with terlipressin was indeed associated with reduced mortality in VS patients less than 60 years old. They also determined that terlipressin may cause peripheral ischemia, albeit while improving renal function.

### Others: Corticosteroids, Vitamins (C, B1, and B12), and Midodrine

Corticosteroid-balancing protocols are commonly used as a coadjutant therapy in the reversion of VS. The practical outcome of these terms is that corticosteroids alone may act upon restoring vascular responsiveness to vasopressors and therefore aid in re-establishing blood pressure in VS. The mechanisms involved in this effect may involve multiple pathways, both genetic and non-genetic, by increasing endothelium α-adrenergic receptor gene expression and by inhibiting inflammation, respectively.^19^

Vitamin C shares many properties with corticosteroids, including activation of nuclear factor-kB, downregulation of proinflammatory mediators, preservation of endothelial function and tightening of their junctions with epithelial cells, protection of the microcirculation, acting as an essential cofactor for catecholamine synthesis, and stimulating the expression of vasopressor receptors. Also, vitamin C is an efficient scavenger of free radicals, preventing additional tissue damage and even more intense inflammatory response and simultaneously, preventing or even reversing the oxidation of corticosteroid receptors and maintaining proper endothelial response to these hormones.^20^

Another vitamin has recently emerged as an alternative in the treatment of VS. The synthetic analog of vitamin B12, hydroxocobalamin, is FDA-approved only for its traditional use as a chelating agent for cyanide poisoning, which results in the production of cyanocobalamin that can be renally excreted. For a recent and thorough review of hydroxocobalamin in the treatment of vasoplegia, we suggest referring to Shapeton et al.^21^

Like phenylephrine and dopamine, midodrine is a selective α1-adrenergic agonist and has been recently drawing attention as a potential alternative adjunctive treatment to VS, mostly by its oral posology. Presently, midodrine treatment is the only FDA-approved for symptomatic orthostatic hypotension, and therefore, its use in VS is off-label.^8^

### Methylene Blue

Methylene blue inhibits guanylate cyclase, lowers Cyclic guanosine monophosphate (cGMP) production, and has been used successfully as a treatment for vasopressor-refractory septic shock vasoplegia. The supposed MB mechanism is the inhibition of the microvasculature by endothelial nitric oxide and improved responsiveness to amines. In this environment, the main question arises: What can we do when circulatory shock becomes refractory to classical therapeutic measures, including administration of fluids, inotropes, and vasoconstrictors? The answers to this question are currently limited to the accumulated evidence regarding 3 cAMP-independent vasoconstriction mechanisms: (1) cGMP/NO-dependent vasoconstriction (the most important mechanism), (2) vasopressin administration, and (3) hyperpolarization-dependent vasoconstriction. Why do not these therapeutic alternatives always work? We believe that there are at least 5 aspects to this investigation: (1) lack of consideration of existing guidelines or evidence-based medicine about the accepted treatment options available, (2) the lack of more excellent knowledge of the different vasodilation mechanisms, (3) the possibility of interference between different vasodilation mechanisms; (4) the enzymatic activity of soluble guanylyl cyclase (sGC), and (5) the frequent use of MB as a therapeutic “rescue” or “final” attempt.^13^

## Emergent Physiopathological Concepts and Treatment Options

### Broad-Spectrum Vasopressor Concept

Like the variable responsiveness of anti-microbial sensitivity present in the clinical approach, vasopressors may provide distinct physiological outcomes. Apart from the first-line treatment with norepinephrine, when refractory shock occurs, various physicians choose to employ a second catecholamine vasopressor, such as epinephrine. However, this approach may not be a logical association, since both vasopressors target the same receptors, congesting the same pathway and yielding little to no positive response. This situation may lead to catecholamine toxicity, leading to a worse prognosis. Sometimes, clinicians may choose to use non-catecholamine vasopressors (vasopressin, angiotensin II) to restore blood pressure by activating complementary pathways.

### Catecholamine Vasopressor Support Sparing Strategies concept

The catecholamine vasopressor supporting sparing strategies concept lies in the need to achieve the target blood pressure without employing an excess of catecholamines, therefore preventing irreversible damage to the microcirculation and cardiocirculatory failure. Recently, the literature has proposed a stream of larger studies regarding MB in refractory shock. Although fluids and amines are indisputable in keeping cardiovascular pressures enough for organ perfusion, the exclusive reliance on this doctrine leads to inevitable microcirculatory injury and the central problem of cardiocirculatory irreversibility.

### Microcirculatory Protection

Microcirculation protection is a fundamental component of hemodynamic stabilization, which goes hand in hand with fluid resuscitation and pharmacological intervention. Clinically, the microcirculation's health is most commonly assessed through the sublingual microvasculature, which was previously thought to be a good surrogate of the microcirculation's general state.

The latest edition of SSC guidelines recommends the “hour-1 bundle,” the infusion of 30 mL/kg aiming for a MAP of at least 65 mmHg and the NE infusion, in case this goal is not achieved in the first hour. Not only reaching this threshold is important, but the sooner this happens and becomes steady, the more negatively correlated are the mortality rates. However, more recent studies bring to attention the dissociation between the monitoring of macroscopic hemodynamic parameters, such as the MAP, and the state of the microcirculation. Optimal MAP values should be individualized according to previous comorbidities and age. Such discussion is becoming increasingly meaningful, and it is time to challenge the status of MAP as a usual surrogate of global perfusion pressure. Several organs present the physiological autoregulation, the ability to maintain a constant influx of blood flow, as long as perfusion pressure remains within the range of the “autoregulation zone.” However, this safe range varies among different organs, with the kidney presenting the highest threshold and should be the first target of reperfusion. We guess that combining these 3 concepts will be useful for better results against the high rates of mortality in critically ill patients. Currently, new theoretical propositions are being discussed. The “broad-spectrum vasopressors” is an emerging concept, considering the drug's associations with diverse pharmacological mechanisms (membrane receptors, endothelium-dependent mechanisms...), adopting “vasopressor support sparing strategies.” These protocols do not have to be considered “rescue” therapy; however, a precocious window of opportunity is essential. Search for novel vasopressor agents, such as synthetic human angiotensin II, which would increase blood pressure and reduce the need for high doses of catecholamine vasopressors. Optimistically, if possible, seek new vasopressors that increase arterial blood pressure without causing microcirculatory damage.
